# The acromegaly patient experience: burden of treatment and quality of life

**DOI:** 10.1210/clinem/dgag122

**Published:** 2026-04-22

**Authors:** Eliza B Geer, Jill Sisco

**Affiliations:** Departments of Medicine and Neurosurgery, Multidisciplinary Pituitary and Skull Base Tumor Center, Memorial Sloan Kettering Cancer Center, New York, NY 10021, USA; Acromegaly Community, Grove, OK 74344, USA

**Keywords:** acromegaly, quality of life, patient-reported outcomes, symptom burden, medical therapy

## Abstract

Acromegaly is a chronic multisystem disorder in which growth hormone and insulin-like growth factor 1 excess cause progressive somatic, metabolic, psychological, and functional morbidity. Although biochemical control improves outcomes, many patients continue to experience persistent symptoms, impaired health-related quality of life (HRQoL), and substantial treatment burden. This review synthesizes data from clinical trials, longitudinal cohorts, registry studies, and patient-reported outcome (PRO) research evaluating physical symptoms, HRQoL, mood, interpersonal functioning, work productivity, and financial burden in acromegaly. We examine validated PRO instruments and the impact of medical, surgical, and radiation therapies on the patient experience. Fatigue, musculoskeletal pain, arthropathy, sleep disturbance, and body-image concerns are highly prevalent and frequently persist despite biochemical remission. HRQoL remains impaired in physical, psychological, and social domains, with depression and anxiety affecting a substantial proportion of patients. Treatment-related factors, including injection burden, breakthrough symptoms, gastrointestinal effects, and financial and surveillance demands further reduce well-being and productivity. PRO tools, including the Acromegaly Quality of Life Questionnaire, Patient-Assessed Acromegaly Symptom Questionnaire, Acromegaly Treatment Satisfaction Questionnaire, and the Acromegaly Symptom Diary, reveal discordance between biochemical control and PROs, highlighting the need for standardized PRO assessment and validated minimal important difference thresholds. New oral therapies and long-acting formulations may reduce treatment burden, but comparative PRO data are limited. Despite therapeutic advances, acromegaly remains associated with considerable symptom burden and impaired HRQoL. Patient-centered care requires systematic PRO incorporation, multidisciplinary management of comorbidities, attention to treatment burden, and shared decision-making.

Acromegaly is a rare endocrine disorder characterized by sustained excess growth hormone (GH) secretion and elevated insulin-like growth factor 1 (IGF-I) levels, typically due to a pituitary adenoma. This chronic, multisystem condition is associated with comorbidities and physical changes related to GH/IGF-I excess, adenoma mass effects, and adverse treatment effects. These include soft-tissue enlargement, musculoskeletal conditions, hypertension, insulin resistance, obstructive sleep apnea, cardiovascular disease, increased cancer risk, and a myriad of physical and psychological symptoms, all of which contribute to disease burden and lead to impaired quality of life (QoL) (1, 2).

Treatment success is traditionally determined by controlling serum IGF-I levels, minimizing adenoma burden, and managing comorbidities. However, patients often prioritize achieving functional remission, that is, clinical recovery in the personal, professional, and social domains. Severity of patients’ symptoms does not always correlate with biochemical status, and the patient perspective of persistent disease activity may differ from that of their physician (3). The patient experience, as assessed by patient-reported outcomes (PROs), is therefore an essential metric for comprehensive assessment of disease status.

This narrative review summarizes literature on the patient experience across the chronic acromegaly journey, including physical symptoms, health-related quality of life (HRQoL), mood, interpersonal and social well-being, work productivity, financial well-being, and strategies to improve patient experience and HRQoL outcomes. Relevant English-language articles were identified through targeted PubMed searches using terms including *acromegaly*, *patient experience*, *quality of life*, *patient-reported outcomes*, *treatment burden*, *breakthrough symptoms*, *mood*, *social well-being*, *work productivity*, and *financial burden*, with additional studies identified through reference list review. Evidence was synthesized qualitatively with emphasis on patient-reported symptoms, functional impact, treatment satisfaction, and opportunities to improve care.

Potential bias related to conflicts of interest should be considered in interpreting this review, particularly for sections discussing medical therapy treatment burden, symptom control, and patient experience, where available evidence may include industry-sponsored studies. In addition, limited data in some domains may increase reliance on expert interpretation and selective reporting within the published literature.

## Use of PROs to assess the acromegaly patient experience

PROs pertain to a patient's health, QoL, or functional status (associated with health care or treatment) that are reported directly by the patient, without interpretation by a clinician (4). PROs and HRQoL measures are central to the evaluation of disease burden and treatment efficacy in acromegaly. Several tools have been developed and validated to assess the patient experience accurately and comprehensively, which include PROs as well as physician-administered assessments.

### Disease-specific PROs

The most widely used PRO tools in acromegaly are the Acromegaly Quality of Life Questionnaire (AcroQoL) and the Patient-Assessed Acromegaly Symptom Questionnaire (PASQ). AcroQoL assesses physical, psychological, appearance, and personal relations domains with 22 questions (scores 22 worst to 110 best) (5). The PASQ, developed to monitor acromegaly symptom severity in response to treatment (6-8), quantifies 6 symptoms (soft-tissue swelling, arthralgia, headache, hyperhidrosis, paresthesia, and fatigue) measured on a scale of 0 to 8, with a higher score (up to 40) indicating greater symptom burden. Newer instruments include the Acromegaly Treatment Satisfaction Questionnaire (Acro-TSQ) and the Acromegaly Symptom Diary (ASD). The Acro-TSQ, designed for patients receiving injectable somatostatin receptor ligands (SRLs), captures symptom interference, treatment convenience, injection site reactions, gastrointestinal interference, and emotional impact (9-11). The ASD assesses symptom severity (12), including 7 core symptoms (headache, joint pain, sweating, fatigue, leg weakness, swelling, numbness/tingling) plus additional items for sleep difficulty and short-term memory difficulty. Patients rate symptoms for the previous 24 hours from 0 (no symptom) to 10 (worst symptom), with a score range from 0 to 70. The ASD is unique in that it allows for daily assessment of symptoms and captures fluctuations in real time during a treatment cycle (12).

### Minimal important difference

The minimal important difference (MID) is the smallest change in a PRO score perceived as beneficial by patients. For AcroQoL, a universally accepted MID has not been established, but changes of 10 points or more are considered clinically meaningful based on distribution and anchor-based methods and observed effect sizes in longitudinal studies (9, 13, 14). For PASQ, no formal MID exists, but standardized mean differences (SMDs) of 0.3 to 0.9 for individual symptoms are interpreted as moderate to large effects, with a mean decrease of −2.3 points (95% CI, −1.3 to −3.3) during treatment (9). Acro-TSQ MID thresholds are validated for key domains: symptom interference (10-12), treatment convenience (9-11), and gastrointestinal interference (8-11). MID thresholds provide a benchmark for interpreting the clinical relevance of PRO changes with treatment and, therefore, contain essential information about treatment responses not captured by assessment of statistical significance of the overall cohort's mean change, as commonly reported in clinical trials.

### Generic instruments

The Short Form (SF)-36 and SF-12 health survey, EuroQol 5 dimension (EQ-5D), psychological general well-being schedule (PGWBS), and patient-reported outcomes measurement information system (PROMIS) are used to assess broader domains of health. SF-36 and SF-12 assess physical functioning, vitality, mental health, and social functioning, while EQ-5D provides a utility index and visual analog scale for overall health status. PROMIS, though not yet applied to acromegaly, uses fixed-length or adaptive questionnaires to assess physical, mental, and social health across diseases, tailoring items for precision and low burden, and generating scores standardized to US population norms for cross-population comparisons (15). Additional domain-specific instruments include the Beck Depression Inventory (BDI), hospital anxiety and depression score, and state-trait anxiety index.

### Clinician-reported tools

Concordance between physician and patient-reported treatment outcomes is low for acromegaly (3). Both patient- and clinician-reported tools are recommended to capture the spectrum of disease impact and guide individualized care (16). Clinician-reported outcome tools (SAGIT; signs/symptoms, associated comorbidities, GH levels, IGF-I levels, and tumor profile; acromegaly disease activity tool; and ACROSCORE) provide structured assessments of disease activity, symptom burden, and treatment response, and discriminate between controlled and uncontrolled disease states (17, 18).

#### Unmet needs in PRO data

Despite substantial progress in evaluating PROs in acromegaly, the use of validated PRO instruments is inconsistent and heterogeneous among reported clinical trials (Table 1) (9-12, 14, 17, 19-83), limiting the ability to compare outcomes to established MID thresholds. Direct head-to-head studies comparing patient-reported symptom improvement or QoL between different medical therapies have rarely been reported. Consistent use of a comprehensive set of PRO tools with established MIDs, including generic, disease-specific, and domain-specific assessments, is an unmet need. Standardized use of a comprehensive set of validated PROs would allow for comparisons between studies and therapies. A comprehensive core outcome set should ideally be developed, validated, and included in acromegaly clinical trials.

**Table 1:** Review of select validated patient-reported outcomes questionnaires in acromegaly studies

Patient reported outcome assessments	Validated*^a^*	Normative US data available	Number of acromegaly studies using assessment*^b^*	Domains assessed	Clinical utility	Scoring (clinical cutoff or minimal important difference, if available)
**Generic QoL assessments**						
EuroQol 5 dimension (EQ-5D)	Yes (19)	Yes (20)	13	Mobility, self-care, usual activities, pain/discomfort, and anxiety/depression plus a VAS for overall self-rated health (19)	Broad clinical utility as a standardized, generic measure of HRQoL; widely used in clinical practice, research, and health economics; less sensitive to acromegaly-specific changes (19, 21)	Utility score −1 to +1, where 0 = death, 1 = perfect health (22) EQ-VAS, 1-100, higher score = QoL (19) Currently no consensus on MID (23)
General Health Questionnaire 30-Item (GHQ-30)	Yes (24)		1	General illness, somatic symptoms, sleep disturbance, social dysfunction, anxiety and dysphoria, suicidal depression (25)	Designed to screen nonpsychotic psychiatric disorders (25)	Score 0-5 for each subscale (30 total), higher score = more severe (25) No established MID
Kellner's Symptom Questionnaire (KSQ)	Yes (26)		1	Psychological distress (anxiety, depression, somatization, hostility-irritability) and well-being (relaxation, contentment, physical well-being, friendliness) (26)	Highly sensitive in detecting psychological distress or impairment in well-being (26)	Score 0-68 for distress subscale, 0-24 for well-being subscale, higher score = worse QoL (26) Cutoff for moderate distress = 1-2 SD above normal Cutoff for severe distress = 2 SD above normal (26) No established MID
Nottingham Health Profile (NHP)	Yes (27)		4	Emotional reactions, energy, pain, physical mobility, sleep, social isolation (27)	Domain scores can inform clinical decision-making by highlighting areas of significant patient distress (27)	Score 0-100, higher score = worse QoL (28) No established MID (29)
Psychological General Well-Being Schedule (PGWBS)	Yes (30)	Yes (31)	3	Anxiety, depression, positive well-being, self-control, general health, vitality (22)	Comprehensive assessment of psychological well-being (30)	Score 0-110, higher score = better well-being (22) MID = > 50% baseline SD for corresponding baseline mean QoL score (22)
Short Form-12 (SF-12) (abbreviated version of Short Form-36 [SF-36])	Yes (32)	Yes (32)	3	Same as SF-36, but with only 1-2 items per domain (32)	Shorter, quicker version of the SF-36; only generates PCS and MCS; advantage over SF-36 to use in large trials (32)	For PCS and MCS, norm-based scoring, with mean (SD) of 50 (10) for the general population, higher score = better QoL (32) MID ≈0.5 SD in chronic disease (33)
SF-36	Yes (28)	Yes (34)	41	Bodily pain, general health perceptions, general mental health, physical functioning, role limitations due to emotional problems, role limitations due to physical health, social functioning, vitality (28, 35)	Allows for broad comparisons across diseases, treatments, and populations; standardized scoring facilitates interpretation with normative data (34) SF-36 provides more reliable estimates of individual levels of health over SF-12 (32)	Score 0-100, higher score = better QoL (28) MID ≈0.5 SD in chronic disease (33) Clinically relevant change in SF-36 scores not known for pituitary patients (36)
World Health Organization Quality of Life-Brief (WHOQoL-BREF)	Yes (37)	Yes (38)	3	Environment, physical health, psychological health, social relationships (37)	Practical tool for holistic QoL assessment, monitoring, and research in a wide range of clinical and cultural contexts (37, 39)	Score 0-100, higher score = better QoL (40) Cutoffs vary by population/condition (41) No established MID (29)
**Acromegaly-specific QoL assessments**						
Acromegaly Comorbidity and Complaints Questionnaire (Acro-CQ)	Yes (42)		2	Comorbidities associated with acromegaly and its treatment; family history of pituitary adenoma (42)	Inexpensive, reliable tool for rapid, systematic collection of clinical data in acromegaly at diagnosis and follow-up (42)	No established MID
Acromegaly Symptom Diary (ASD)	Yes (12)		2	Core symptoms (fatigue, headache, joint pain, leg weakness, sweating, swelling, numbness/tingling), sleep difficulty, short-term memory difficulty (12, 43)	Acromegaly-specific PRO developed based on FDA guidance for use in acromegaly clinical trials (12)	Score 0-70, higher score = worse QoL (12, 43) MID = 4-6 point change (12)
Acromegaly Treatment Satisfaction Questionnaire (Acro-TSQ)	Yes (11)		3	Symptom and gastrointestinal side effect interference, treatment satisfaction, treatment bother, treatment convenience (11)	Developed specifically for patients receiving injectable somatostatin receptor ligands (SRLs) (10, 11) Assesses aspects of disease and treatment that patients consider important, relevant, and impactful (10)	Score 0-100, higher score = highest satisfaction/lowest interference (11) MIDs: Symptom interference = 10-12 points, treatment convenience = 9-11 points, gastrointestinal interference, 8-10 points (11)
Acromegaly Quality of Life Questionnaire (AcroQoL)	Yes (19)		81	Physical and psychological (appearance and personal relationships) function (19, 44)	Designed specifically for use in clinical trials and routine monitoring of patients with acromegaly (44) Changes ≥10 points likely meaningful (14)	Score 0-110, higher score = better QoL (19) Meaningful improvement global score ≥ responder threshold (increase ≥50% of baseline SD of score) (45)
Leiden Bother and Needs Questionnaire—Pituitary (LBNQ-Pituitary)	Yes (46)		1	Issues in sexual functioning, Issues in social functioning, mood problems, negative illness perceptions, physical and cognitive complaints (46)	Assesses degree to which patients are bothered by the consequences of pituitary disease and their needs for support (46)	Score 0-100, higher score = greater need for support (46) MID = 0.5 SD between timepoints (47)
Patient-Assessed Acromegaly Symptom Questionnaire (PASQ)	No (48)		17	Symptoms (headache, perspiration, joint pain, fatigue, soft tissue swelling, numbness or tingling of limbs) and overall perceived health (49)	Evaluates symptoms and treatment success (49) Focuses on most common signs and symptoms of acromegaly (17)	Score 0-58 higher = more severe symptoms (49) SMD = 0.2 small effect, 0.5 moderate effect, 0.8 large effect (9)
**Symptom Specific Assessments**						
Beck Anxiety Inventory (BAI)	Yes (50)	Yes (51)	4	Anxiety (50)	Easily administered, relatively brief, and easily scored measure of anxiety (52)	Score 0-63, higher score = worse anxiety (50, 53, 54) Cutoffs: 10-18 = mild to moderate anxiety, 19-29 = moderate to severe anxiety; 30-63 = severe anxiety (52) No established MID
Beck Depression Inventory (BDI) and BDI-II	Yes (55)	Yes (56)	15	Cognitive, emotional, motivational, physical symptoms in depression (57)	Standardized tool for screening, measuring depression severity, and monitoring treatment progress in clinical and research settings (58, 59)	Score 0-63, higher score = worse depression (57, 60) Cutoffs: 14-19 = mild depression; 20-28 = moderate depression; 29-63 = severe depression (58) MID = 17.5% score reduction; 32% in those with more severe depression (61)
Epworth Sleepiness Scale (ESS)	Yes (62)	Yes (63)	13	General daytime sleepiness (62)	Assesses tendency to fall asleep in 8 different situations (64) Routinely used in evaluating sleep disorders Test-retest variability in clinical settings vs original validation study may limit clinical utility (62)	Score 0-24, higher score = higher levels of sleepiness (62) Cutoff score of 7 indicates increasing degree of excessive daytime sleepiness (65) No established MID
Hospital Anxiety and Depression Scale (HADS)	Yes (46)		6	Anxiety, depression (46)	Practical tool for identifying and quantifying anxiety and depression (28)	Score 0-21, higher score = greater symptoms of depression/anxiety (46) Score >8 on either subscale indicates patients as being anxious or depressed, respectively (46) Scores associated with reduction in perceived HRQoL (28) No established MID
Multidimensional Fatigue Inventory-20 (MFI-20)	Yes (46)		4	General fatigue, mental fatigue, physical fatigue, reduced activity, reduced motivation (46)	Assesses multidimensional aspects of fatigue (66)	Score 0-20, higher score = greater fatigue (46) No established MID
Pittsburgh Sleep Quality Index (PSQI)	Yes (67)	Yes (68)	5	Daytime dysfunction, habitual efficient sleep, sleep disturbances, sleep duration, sleep latency, subjective sleep quality, use of sleeping medications (69)	Designed for clinical assessment (67) Assesses sleep quality over one month interval allows discrimination of transient and persistent sleep disturbances (69)	Score 0-21, higher score = worse sleep quality (69) Cutoff score of 5 distinguishes between good and poor sleepers (67) MID = score reduction of 3 or more (70)
Rosenberg Self-Esteem Scale (RSES)	Yes (71)	Yes (72)	2	Global self-esteem as a single domain (73)	Simple, brief, widely used, no cutoff population norms (73)	Score 0-30 or 10-40, higher score = better self-esteem (71, 73) No established MID
State Trait Anxiety Inventory (STAI)	Yes (74)	Yes (52)	5	State anxiety, trait anxiety (60)	Can be used to diagnose anxiety and differentiate from depressive syndromes Can also evaluate caregiver distress (74)	Score for each subscale 0-60 or 20-80, higher score = greater anxiety (60, 75) Using the 20-80 scoring system, cutoff score of 40 indicates abnormal anxiety (40-59 = moderate anxiety, 60-80 = severe anxiety) (75) No established MID
Western Ontario and McMaster Universities Osteoarthritis Index (WOMAC)	Yes (76)		7	Articular pain, stiffness, functionality (77)	Measures dysfunction and pain associated with osteoarthritis of the lower extremities Widely used in clinical trials (76)	Score for each subscale 0-100 using VAS, higher = worse outcome (78, 79) MID cutoffs vary by condition (80)
Work Productivity and Activity Impairment questionnaire (WPAI)	Yes (81, 82)	Yes (83)	1	Absenteeism, presenteeism, overall work impairment, activity impairment (82)	Enables quantitative assessment of disease impact on paid and unpaid work (82)	Score 0-100%, higher score = greater work impairment (82) No established MID

EQ-5D: (“acromegaly”[MeSH Terms] OR acromegaly[tiab]) AND (“EQ-5D”[tiab] OR “EQ5D”[tiab] OR “EuroQol”[tiab] OR “EuroQol-5D”[tiab] OR “EuroQol five dimensions”[tiab] OR “EuroQoL”[tiab] OR “EQ 5D”[tiab])

GHQ-30: (“acromegaly”[MeSH Terms] OR acromegaly[tiab]) AND (“30-Item General Health Questionnaire” [tiab] OR “GHQ-30”[tiab])

KSQ: (“acromegaly”[MeSH Terms] OR acromegaly[tiab]) AND (“Kellner's symptom questionnaire” [tiab] OR “KSQ”[tiab] OR “Kellner” [tiab])

NHP: (“acromegaly”[MeSH Terms] OR acromegaly[tiab]) AND (“NHP”[tiab] OR “Not**t**ingham Health Profile”[tiab])

PROMIS-29: (“acromegaly”[MeSH Terms] OR acromegaly[tiab]) AND ((“Patient-Reported Outcomes Measurement Information System”[tiab]) OR “PROMIS-29”[tiab] OR “PROMIS 29”[tiab] OR “PROMIS29”[tiab] OR “PROMIS”[tiab]) (note: No studies in patients with acromegaly found in the PubMed title/abstract search and not included in the table)

PGWBS: (“acromegaly”[MeSH Terms] OR acromegaly[tiab]) AND ((“Psychological General Well-Being”[tiab]) OR “Psychological General Well-Being Index”[tiab] OR “Psychological General Well-Being Schedule”[tiab] OR PGWB[tiab] OR PGWBS[tiab])

SF-12: (“acromegaly”[MeSH Terms] OR acromegaly[tiab]) AND (“SF-12”[tiab] OR “Short Form 12”[tiab] OR “Short Form Health Survey”[tiab])

SF-36: (“acromegaly”[MeSH Terms] OR acromegaly[tiab]) AND (“SF-36”[tiab] OR “Short Form 36”[tiab] OR “Short Form Health Survey”[tiab])

WHOQoL-BREF: (“acromegaly”[MeSH Terms] OR acromegaly[tiab]) AND (“WHOQOL-BREF”[tiab] OR “WHOQOL BREF”[tiab] OR “World Health Organization Quality of Life”[tiab] OR “World Health Organization Quality of Life-BREF”[tiab] OR “WHO Quality of Life”[tiab])

Acro-CQ: (“acromegaly”[MeSH Terms] OR acromegaly[tiab]) AND (“Acro-CQ”[tiab] OR “Acromegaly Comorbidity and Complaints Questionnaire”[tiab] OR “ACCQ”[tiab])

ASD: (“acromegaly”[MeSH Terms] OR acromegaly[tiab]) AND (“Acromegaly Symptom Diary”[tiab] OR “ASD”[tiab] OR “Acromegaly Symptom Diaries”[tiab])

Acro-TSQ: (“acromegaly”[MeSH Terms] OR acromegaly[tiab]) AND (“Acromegaly Treatment Satisfaction Questionnaire”[tiab] OR “Acro-TSQ”[tiab] OR “treatment satisfaction questionnaire”[tiab])

AcroQoL: (“acromegaly”[MeSH Terms] OR acromegaly[tiab]) AND (“Acromegaly Quality of Life Questionnaire”[tiab] OR AcroQoL[tiab] OR “Acro QoL”[tiab])

LBNQ-Pituitary: (acromegaly[MeSH Terms] OR acromegaly[tiab]) AND (“Leiden Bother and Needs Questionnaire Pituitary”[tiab] OR “Leiden Bother and Needs Questionnaire”[tiab] OR “LBNQ-Pituitary” [tiab] OR “LBNQ Pituitary” [tiab] OR “LBNQ” [tiab] OR “Leiden Bother & Needs Questionnaire”[tiab] OR “Bother and Needs Questionnaire”[tiab])

PASQ: (“acromegaly”[MeSH Terms] OR acromegaly[tiab]) AND (“Patient-Assessed Acromegaly Symptom Questionnaire”[tiab] OR “PASQ” [tiab] OR “Patient Assessed Acromegaly Symptom Questionnaire”[tiab])

BAI: (“acromegaly”[MeSH Terms] OR acromegaly[tiab]) AND (“Beck Anxiety Inventory”[tiab] OR “BAI” [tiab])

BDI: (“acromegaly”[MeSH Terms] OR acromegaly[tiab]) AND (“Beck Depression Inventory”[tiab] OR “BDI” [tiab])

ESS: (“acromegaly”[MeSH Terms] OR acromegaly[tiab]) AND (“Epworth Sleepiness Scale”[tiab] OR “ESS” [tiab])

HADS: (“acromegaly”[MeSH Terms] OR acromegaly[tiab]) AND (“Hospital Anxiety and Depression Scale”[tiab] OR “HADS” [tiab])

MFI: (“acromegaly”[MeSH Terms] OR acromegaly[tiab]) AND (“Multidimensional Fatigue Inventory”[tiab] OR “MFI” [tiab])

PSQI: (“acromegaly”[MeSH Terms] OR acromegaly[tiab]) AND (“Pittsburgh Sleep Quality Index”[tiab] OR “PSQI” [tiab])

RSES: (“acromegaly”[MeSH Terms] OR acromegaly[tiab]) AND ((“Rosenberg Self-Esteem Scale”[tiab]) OR “Rosenberg self esteem scale”[tiab] OR “Rosenberg Self Esteem”[tiab] OR RSES[tiab])

STAI: (“acromegaly”[MeSH Terms] OR acromegaly[tiab]) AND (“State Trait Anxiety Inventory”[tiab] OR “STAI” [tiab])

WOMAC: (“acromegaly”[MeSH Terms] OR acromegaly[tiab]) AND (“Western Ontario and McMaster Universities Osteoarthritis”[tiab] OR “WOMAC” [tiab])

WPAI: (“acromegaly”[MeSH Terms] OR acromegaly[tiab]) AND (“Work Productivity and Activity Impairment questionnaire”[tiab] OR “Work Productivity and Activity Impairment” [tiab] OR “WPAI” [tiab])

Abbreviations: EQ-VAS, EuroQol Visual Analogue Scale; FDA, US Food and Drug Administration; HRQoL, health-related quality of life; MCS, mental component score; MID, minimal important difference; PCS, physical component score; PRO, patient-reported outcomes; SD, standard deviation; SMD, standardized mean difference; VAS, visual analog scale; QoL, quality of life.

^*a*^Patient-reported outcome assessments were classified as validated if validation was reported in instrument development or early validation studies, or if the instrument was referenced as validated in secondary sources, even when primary validation data were not directly assessed.

^*b*^PubMed Search strings—PubMed was searched using a combination of keywords and medical subject headings (MeSH) terms listed below for each patient-reported outcomes questionnaire in acromegaly. This search was conducted in October–November 2025 and limited to English-language articles published in peer-reviewed journals. These strings were applied with no date limit. Titles and abstracts were reviewed only and did not include reviews or validation studies. No other restrictions were applied.

## Physical symptoms

### Prevalence and severity

Fatigue, muscle weakness, and joint pain are the most bothersome physical complaints that impact daily functioning and are reported in 79% to 92% of patients (84-86). Increased hand and foot size, frontal bossing, prognathism, and soft-tissue swelling are reported in 83% to 88% of patients at diagnosis (1, 84, 87). Hyperhidrosis and seborrhea affect up to 80% of patients with acromegaly, and obstructive sleep apnea affects 65% to 81%, and carpal tunnel syndrome affects 64%. Headache is reported in 55% to 60% of patients (85).

Musculoskeletal conditions affect >80% of patients with acromegaly and contribute to impaired QoL, disability, and health care resource utilization (88-93). Arthropathy, present in ∼70% of patients at diagnosis, along with polyarticular arthritis, osteophyte formation, and vertebral fractures, frequently causes disabling pain and functional limitation (94).

A Danish registry study that included 844 patients with acromegaly matched 1:100 with healthy controls (95) found an increased risk of shoulder and knee osteoarthritis, even before diagnosis, along with higher rates of joint replacement and postoperative complications. Osteoarthritis continued to progress over time even after treatment. Axial arthropathy was also more prevalent, with a 2-fold increased risk of spondylosis, spinal stenosis, and back pain, and an 80% increased risk of vertebral disc herniations. Musculoskeletal disorders have been associated with more analgesic prescriptions, including for opiates: 25% of patients with acromegaly received ≥1 opioid prescription (95), and 31% reported regular use of analgesics (28).

Acromegaly is associated with high-turnover osteoporosis with compromised bone microarchitecture leading to increased vertebral fracture risk, often despite normal bone mineral density (96, 97). Hypogonadism, present in ≥20% of patients, exacerbates fracture risk. In the same registry study (95), patients had a >2-fold increased risk of osteoporosis. Some studies show fewer vertebral fractures after treatment (95), whereas others report continued risk despite remission (98, 99).

### Determinants of physical symptoms

Diagnostic delay contributes to symptom burden, as progression of physical symptoms occurs over time in the setting of elevated GH/IGF-I levels. Arthropathy is irreversible, even after biochemical control, defined as a serum IGF-I within the age-stratified normal range. Women consult physicians more frequently and present with acromegaly at an older age at least in part due to diagnostic delays (2-4.6 years longer than their male counterparts) (100, 101). Longer time to diagnosis in women may in part be due to differences in symptom recognition or health care-seeking behavior (100, 102, 103). Consequently, women typically present with more comorbidities and report worse physical functioning and QoL (101, 104, 105). Men tend to present at a younger age and with larger, more invasive adenomas, which are associated with more pronounced acral and soft tissue changes (100).

Disease severity, reflected by GH and IGF-I levels, adenoma size, and duration of untreated disease, is a major determinant of symptom burden. Higher GH and IGF-I levels are associated with more severe soft tissue swelling, fatigue, joint pain, and metabolic and cardiovascular complications (1, 106). Early intervention and effective control of GH/IGF-I levels are critical to limit progression of arthropathy, but joint complaints and musculoskeletal pain may persist despite biochemical control, reflecting irreversible structural changes (85, 107).

### Impact of acromegaly treatments on physical symptoms

The extent to which treatment alleviates symptom burden depends on biochemical efficacy and symptom reversibility (Table 2) (9, 22, 78, 85, 106-119). Surgery can provide rapid relief of symptoms related to mass effect, such as visual compromise and headache, and can improve soft-tissue swelling, sleep apnea, and cardiovascular function, when biochemical control is achieved (120). However, at least half of the patients require multimodal treatment to achieve remission.

**Table 2:** Symptom prevalence in untreated and treated patients with acromegaly

Symptom/domain	Prevalence in untreated disease	Prevalence in treated disease	Nature/impact in treated disease
Fatigue	Fatigue/tiredness 0% to 82% (weighted mean, 53%) (108)	Fatigue/weakness/tiredness 55% to 73% (106, 109)	Improves but may persist, impairs ADLs/QoL (9, 22, 109-111)
Muscle weakness/dysfunction	Asthenia/decreased vigor, 7% to 100% (weighted mean, 22%) (108) 38% (112)	47% (112)	Persistent, functional impairment (78, 110, 113)
Joint pain/arthropathy/arthralgia	7% to 85% (weighted mean, 34%) (108)	28% to 77% (85, 106, 109, 114)	Improves but may persist (85, 106, 107, 109, 114), radiographic progression despite symptom improvement (107, 115), impacts QoL (9, 111)
Acral enlargement/soft tissue swelling	44% to 100% (weighted mean, 90%) (108)	76% (106, 109)	Partial improvement, not always resolved (9, 85, 106, 109)
Headache	0% to 88% (weighted mean, 59%) (108)	38% to 70% (106, 109)	Partial improvement (9, 85, 106, 109)
Hyperhidrosis	5% to 92% (weighted mean, 47%) (108)	43% to 57% (106, 109)	Partial improvement (9, 85, 106, 109)
Anxiety/depression	Depression 5% to 26% (weighted mean, 22%) Anxiety 1% (108)	36% (116)	Persistent, impacts psychosocial well-being (22, 110, 116, 117)
Visual disturbances	6% to 64% (weighted mean, 30%) (108)	49% (109)	Persistent (109)
QoL impairment	Severe (22, 117, 118)	Persists (85, 116, 117, 119), with symptoms interfering with daily life (92%), leisure activities (84%), and work activities (87%) (118)	Physical function, appearance, psychosocial domains (9, 22, 110, 116, 118, 119)
Treatment side effects	Not applicable	74–77% (118)	Interferes with daily life (116, 118)

Abbreviations: ADL, activities of daily living; QoL, quality of life.

Radiation therapy induces remission over 5 to 10 years in about half of patients; the time to symptom resolution is prolonged, and the potential development of hypopituitarism may contribute to additional symptoms (121-123). Medical therapy results in less complete symptom improvement, and many patients experience persistent physical symptoms despite biochemical control (124).

Two-thirds of biochemically controlled patients on SRLs continued to experience acromegaly symptoms, with 82% reporting symptoms “all of the time” (118), including fatigue, muscle weakness, joint pain, soft tissue swelling, headache, and hyperhidrosis. Arthropathy is refractory, with joint complaints persisting in ∼77% of patients with biochemical remission and radiographic progression of osteoarthritis observed in most controlled patients over nearly a decade of follow-up (85, 107).

Patients with controlled acromegaly exhibit intramuscular (IM) fatty infiltration, slower gait speed, and poorer functional performance, with these deficits associated with reduced QoL across physical, psychological, and social domains (78, 113). Headache, excessive perspiration, and soft tissue swelling improve with treatment but may not resolve completely (9, 106).

Overall, meta-analyses and prospective studies show that while symptom burden improves, patients continue to experience ongoing physical impairment after treatment, as shown by Acro-QoL and PASQ scores (9, 125). Fatigue and musculoskeletal complaints are the least likely to resolve (9, 126).

## HRQoL impairment and mood disorders

### Prevalence and severity

**Figure 1:**
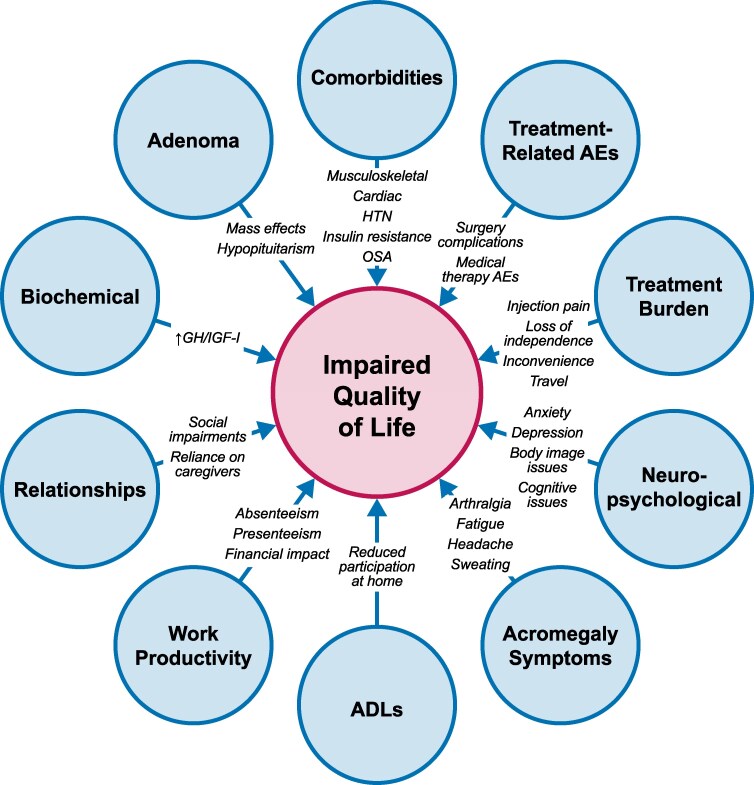
Factors shaping quality of life for patients with acromegaly. ADL, activities of daily living; AE, adverse event; HTN, hypertension; OSA, obstructive sleep apnea; GH, growth hormone; IGF-I, insulin-like growth factor 1.

HRQoL encompasses personal, professional, and social domains and is informed by physical and psychological symptoms, bodily limitations, interpersonal relationships, mood, and capacity to engage socially and professionally (Fig. 1). Patients with acromegaly experience markedly impaired HRQoL at diagnosis, with AcroQoL scores typically ∼57% to 69% of normative values, and SF-36 scores similarly reduced (19, 22, 116, 125, 127). Mood disturbances are common and clinically significant. Depression affects ∼28% to 35% of patients, while anxiety symptoms are reported in up to 66% of patients, exceeding rates in other chronic somatic diseases (77, 117, 128-131). Using the Composite International Diagnostic Interview, a lifetime prevalence of affective disorders of 35% was observed, compared with 21% in patients with other chronic somatic disorders and 11% in healthy controls (131).

### Determinants of HRQoL and mood

Diagnostic delay is a key determinant of impaired HRQoL. Retrospective and cross-sectional studies demonstrate that a longer interval between symptom onset and diagnosis is associated with worse SF-36 and higher BDI-II scores (132). Both the number of physicians consulted and total diagnostic delay independently predict poorer HRQoL and higher depression scores (132), and persistence of psychological distress despite biochemical remission underscores the need for early recognition and intervention (102, 130).

Musculoskeletal complications, metabolic disorders, sleep disturbances, and cardiovascular disease are independently associated with impaired HRQoL and increased depression and anxiety (116, 119). The number and severity of comorbidities at follow-up predict reduced HRQoL, regardless of biochemical control (116, 119). For example, higher HbA1c and body mass index correlate with poorer physical and social domain HRQoL scores (133). Joint pain and arthropathy are particularly linked to psychiatric morbidity, including daily opioid use and higher depression risk (129). These findings highlight the multifaceted impact of comorbidities on psychological and functional outcomes.

Phenotypic changes, fatigue, headache, paresthesia, hyperhidrosis, and soft tissue swelling also contribute to mood disorders and impaired HRQoL (19, 77, 117). The appearance/self-esteem domain of AcroQoL is consistently the most affected, reflecting body image concerns that are associated with lower HRQoL and more depression and anxiety (9, 19, 22, 105, 117, 125). Ongoing physical discomfort and appearance concerns likely contribute to persistent QoL deficits after treatment (9, 125).

Younger patients and those with shorter disease duration report more severe depression, anxiety, and body image distortion, while older patients and those with longer disease duration experience greater cognitive impairment and sexual dysfunction (117, 132). Women consistently report lower physical HRQoL and greater psychological discomfort, particularly in relation to body image and sexual function (77, 116, 117, 125, 134).

### Impact of acromegaly treatments on mood and HRQoL

Specific interventions influence QoL in patients with acromegaly (Table 3) (7, 25, 54, 106, 126, 135-156). A meta-analysis of 46 longitudinal studies found that HRQoL (assessed by AcroQoL and PASQ), paresthesia, hyperhidrosis, fatigue, arthralgia, headache, and soft tissue swelling improved with treatment (9). Nevertheless, longitudinal data show persistent deficits even after biochemical control (Table 3) (22, 125, 127). For example, 58% scored below the 25th percentile on at least one SF-36 domain, and 32% scored below the 25th percentile on 4 or more domains, after 15 to 20 years of sustained biochemical control (86, 116, 127). The appearance/self-esteem domain is consistently the most affected, reflecting the profound impact of somatic disfigurement on psychological well-being (19, 22).

**Table 3:** **Specific interventions influencing quality of life in patients with acromegaly (Adapted with permission from Gadelha MR, et al** (2)

Reference, year of publication	Study design, N, duration of follow-up	Disease status	Intervention	QoL scale	Therapy effect*^a^*
Pharmacotherapy					
Sonino et al 1999 (135)	Prospective, open label, N = 10, 8 weeks	Active, after surgery and radiotherapy and OCT-LAR or BRC	Lanreotide (slow release)	KSQ, CSKSLPP	2+
Biermasz et al 2003 (136)	Prospective, N = 14, 36 weeks	Controlled on OCT-LAR	OCT-LAR: increasing dose interval from 4 to 6 weeks	NHP	0
Neggers et al 2008 (7)	Prospective double-blind, crossover RCT, N = 20, 36 weeks	Controlled on SRL	Addition of weekly PEG vs placebo	AcroQoL, PASQ	2+ (PEG group)
Ghigo et al 2009 (137)	Prospective, randomized, open-label, N = 113, 48 weeks	Active, medical treatment and RT naive	OCT-LAR vs PEG	AcroQoL, Acromegaly signs and symptom scores	2+ (both groups)
Lombardi et al 2009 (138)	Prospective, open label, N = 51, 52 weeks	Active, treatment naive, or post-surgery	Lanreotide Autogel	NHP	2+
Trainer et al 2009 (139)	﻿Prospective randomized, open label, N = 27, 40 weeks	﻿Active, on OCT-LAR	﻿PEG monotherapy vs addition of PEG to SRL	﻿AcroQoL, EQ-5D	﻿2+ (both groups)
Schopohl et al 2011 (140)	Prospective, open label, N = 37, variable (26-52 weeks)	Controlled on OCT-LAR	Switch to lanreotide Autogel varying intervals	AcroQoL	0
Madsen et al 2011 (141)	Prospective, randomized, N = 18, 24 weeks	Controlled on SRL	Continuation of SRL vs addition of PEG	EQ-5D, PASQ	0 (both groups)
Mangupli et al 2014 (142)	Retrospective, observational, N = 28, 208 weeks	Variable control on SRL	OCT-LAR	AcroQoL	2+
Chin et al 2015 (143)	Prospective, open label, N = 58, 24 weeks	Treatment naive	OCT-LAR	AcroQoL	1+
Bronstein et al 2016 (144)	Prospective, open label, N = 119, 52 weeks	Active, on OCT-LAR or PAS-LAR	Cross-over PAS-LAR vs OCT-LAR	AcroQoL	0
Caron et al 2016 (145)	﻿Prospective, open label, N = 90, 48 weeks	Treatment naive	Lanreotide autogel	AcroQoL, PASQ	2+
Dal et al 2018 (146)	Prospective, randomized, open label, N = 61, 52 weeks	Controlled after surgery/on OCT-LAR	SRL titrated according to IGF-I vs GH	AcroQoL, PASQ	0 (both groups)
Colao et al 2019 (147)	Prospective, open label, N = 70, 8 months	Inadequately controlled on conventional SRLs	OCT-LAR vs OCT-LAR + PEG vs OCT-LAR + cabergoline	AcroQoL	0 (all groups)
Coopmans et al 2020 (148)	Prospective, N = 54, 9 months	Controlled on SRL and PEG	Switch to PAS-LAR with or without PEG	﻿AcroQoL, PASQ	2+ (AcroQoL) 1+ (PASQ)
Bolanowski et al 2021 (149)	Prospective, observational, real-world, N = 128, median 13.4 months since treatment initiation	Controlled or uncontrolled on lanreotide autogel	Lanreotide autogel managed within routine care	AcroQoL	1+
Stormann et al 2021 (150)	Prospective, observational, real-world, N = 51, 24 months	Controlled or uncontrolled on lanreotide autogel	Noninterventional	AcroQoL	0
Salvatori et al 2022 (126)	Prospective, open-label, real-world, N = 544, 5 years for QoL assessments	Controlled on PEG or treatment naive/semi-naive	Noninterventional	AcroQoL, PASQ	Numerical improvement*^b^* (AcroQoL, assessed for naive group only) Numerical improvement*^b^* (PASQ, both groups)
Fleseriu et al 2023 (151)	Prospective, open-label, real-world, N = 60, 0 to >3 years	Controlled or uncontrolled on oral OCT or injectable SRL	Continue oral OCT or switch from injectable SRL to oral OCT	Acro-TSQ	Maintained improvement; subscale 2+ (treatment satisfaction, treatment convenience in continued group) Substantial numerical improvement (switch group)
Gandhi et al 2025 (152)	Prospective, open label, nonrandomized, N = 10, 6 months	Controlled modest IGF-I elevation despite SRL with or without PEG	Addition or dose escalation of PEG	AcroQoL, PASQ, ACRODAT	0 (AcroQoL) 2+ (PASQ and ACRODAT)
Pituitary surgery					
Karaca et al 2011 (153)	Prospective, N = 22, 52 weeks	Treatment naive	Surgery vs OCT-LAR	AcroQoL	0 (both groups)
Milian et al 2013 (154)	Prospective, N = 29, 12, and 52 weeks	Treatment naive	Surgery	AcroQoL, SF-36	2+
Fujio et al 2017 (155)	Prospective, N = 41, 52 weeks	Treatment naive	Surgery	SF-36	1+
Ishikawa et al 2019 (25)	Prospective, N = 39, 6 months	Treatment naive	Surgery	SF-36, GHQ-30	0
Gu et al 2020 (156)	Prospective, N = 151, 6 months	Treatment naive	Surgery	AcroQoL, SF-36	2+
Lin et al 2023 (106)	Prospective, N = 106, 6 months	Treatment naive	Surgery	PASQ	2+
Uysal et al 2025 (54)	Prospective, N = 19, 9 months	Controlled at 9 months after surgery	Surgery	AcroQoL, BDI, BAI	2+ (AcroQoL, BDI) 0 (BAI)

Adapted with permission from Table 7 of Gadelha MR, Kasuki L, Lim DS, Fleseriu M. Systemic complications of acromegaly and the impact of the current treatment landscape: an update. *Endocr Rev*. 2019;40(1):268-332. https://doi.org/10.1210/er.2018-00115 (2).

Abbreviations: ACRODAT, Acromegaly Disease Activity Tool; Acro-TSQ, Acromegaly Treatment Satisfaction Questionnaire; AcroQoL, Acromegaly Quality of Life Questionnaire; BAI, Beck Anxiety Inventory; BDI, Beck Depression Inventory; BRC, bromocriptine; CSKSLPP, Cognitive Scale of Kellner's Screening List for Psychosocial Problems; EQ-5D, EuroQol 5 dimensions questionnaire; GHQ-30, 30-Item General Health Questionnaire; IGF-I, insulin-like growth factor 1; KSQ, Kellner's Symptom Questionnaire; LAR, long-acting release; NHP, Nottingham Health Profile; OCT, octreotide; PAS, pasireotide; PASQ, Patient-Assessed Acromegaly Symptom Questionnaire; PEG, pegvisomant; QoL, quality of life; RCT, randomized controlled trial; RT, radiotherapy; SRL, somatostatin receptor ligand.

^*a*^For therapy effect: 0, no significant correlation with QoL; 1+, positive correlation with a subscale of QoL only; 2+, positive correlation with QoL.

^*b*^Significance not specified in the article.

Literature search conducted:

PubMed using the following string: (“Acromegaly”[Mesh] OR acromegaly[tiab]) AND (“Cabergoline”[tiab]) AND (“Quality of Life”[Mesh] OR “Patient Reported Outcome Measures”[Mesh] OR “quality of life”[tiab] OR “QoL”[tiab] OR “patient-reported”[tiab] OR “health-related quality of life”[tiab] OR “HRQoL”[tiab] OR “AcroQOL” [tiab] OR “PASQ” [tiab] OR “SF = 36” [tiab] OR “EQ-5D” [tiab] OR “NHP” [tiab] OR “KSQ” [tiab]) Reperformed the search string with each of the following treatments: cabergoline, lanreotide, paltusotine, pasireotide, pegvisomant, octreotide, surgery Restricted to 2019 and later Did not include reviews, case studies, cost utility analyses, or cross-sectional studies

#### Transsphenoidal surgery

Transsphenoidal surgery improves HRQoL and mood if remission is achieved. In a prospective study, BDI and AcroQoL scores both improved after remission (54). Another study showed global and domain-specific HRQoL gains after surgery independent of biochemical remission, although appearance, vitality, and mental health remained below population norms, underscoring persistent disease burden even after surgery (156).

#### Radiation therapy

Radiation therapy is associated with adverse HRQoL and mood outcomes, independent of biochemical control. Prior radiotherapy predicts poorer SF-36 mental component, higher BDI-II (132), and impaired QoL as assessed by AcroQoL, PGWBS, EuroQol, and signs and symptoms score (19). This likely reflects both the selection of more aggressive disease and the potential adverse effects of radiotherapy, including hypopituitarism.

#### Medical therapy

The impact of medical therapy on mood and HRQoL in acromegaly is complex, influenced by the specific treatment used, the patient's unique disease characteristics, and whether biochemical control is achieved. The Broersen meta-analysis provides important data on medical therapy outcomes, as 36 of these 46 studies used medical therapy as the main treatment. Significantly improved AcroQoL (mean increase 2.9 points, 95% CI 0.5 to 5.3) and PASQ (mean decrease 2.3 points, 95% CI −1.3 to −3.3) were seen with treatment. Symptoms also improved, with the largest effect seen for paresthesia (SMD −0.9), and moderate effects for hyperhidrosis (−0.4), fatigue (−0.3), arthralgia (−0.3), headache (−0.3), and soft tissue swelling (−0.2). These improvements were observed in both treatment-naive and previously treated patients, with the greatest gains in the first year of therapy (9).

#### Injectable somatostatin receptor ligands

A retrospective study of octreotide long-acting release (LAR) in 28 patients found baseline AcroQoL scores of 53 ± 15, which improved to 70 ± 15 after treatment (*P* < .001). Patients with normalized GH (<2.5 μg/L) and IGF-I values had an average AcroQoL increase of 22 points (*P* = .003), while those with improved but not normalized markers had a 16-point increase (*P* = .008). Severe headaches limited QoL improvement (142).

In the PRIMARYS study, a 1-year, open-label trial of lanreotide in 90 patients, ∼ 60% of patients achieved MID in PASQ total score, and >40% achieved MID in AcroQoL global score. Improvements in HRQoL were greater in patients who achieved biochemical control, but symptom improvements were observed regardless of biochemical status (145). Long-term treatment with lanreotide from the SALSA study showed sustained symptom and QoL improvement up to 8 years (157).

For pasireotide, the phase 3 PAOLA study and its extension reported improvements in headache, fatigue, perspiration, paresthesia, osteoarthralgia, and reductions in ring size over nearly 6 years of treatment, although validated PRO instruments were not used (158). Improvement in headache and other symptoms is seen in real-world studies (159), but not all studies show significant improvement in AcroQoL scores (160). More than half of patients develop hyperglycemia, which may negatively impact QoL (160, 161).

#### GH-receptor antagonist

The ACROSTUDY extension (126) included 544 patients treated with pegvisomant for a mean of 7.8 years. Small improvements in PASQ scores were shown, with no difference between IGF-I-controlled and uncontrolled groups. In treatment-naive/semi-naive patients, PASQ and AcroQoL scores remained similar to baseline up to 1 year, regardless of IGF-I control.

#### Comparative and head-to-head pro studies: pegvisomant and injectable SRLs

Direct head-to-head studies comparing symptom or HRQoL improvement between medical therapies are limited to a few studies comparing SRLs and pegvisomant. A prospective, double-blind, placebo-controlled study found that the addition of weekly pegvisomant to ongoing SRL therapy in patients with normalized IGF-I significantly improved AcroQoL (*P* = .008), AcroQoL physical (*P* = .002), PASQ (*P* = .038), and sweating, soft-tissue swelling, and overall health status (7). Other studies, however, show comparable symptom outcomes between pegvisomant and SRLs. A multicenter, open-label, randomized trial comparing pegvisomant and octreotide LAR in 118 patients found that both treatment groups achieved similar improvements in ring size, acromegaly signs and symptom scores, and AcroQoL total scores (137).

#### Treatment-burden associated with injectable SRLs

Use of injectable SRLs may be an independent adverse predictor of HRQoL, highlighting the multifactorial nature of QoL outcomes, which include ongoing symptoms, comorbidities, and administration-related adverse events (116). Several studies have used Acro-TSQ in cohorts on injectable SRLs to assess symptom interference, treatment convenience, injection site reactions, gastrointestinal interference, treatment satisfaction, and emotional impact (11). Most patients biochemically controlled on injectable SRLs (octreotide or lanreotide) reported persistent symptoms and treatment-related side effects that interfere with daily life, leisure, and work activities (118). A significant proportion (43-70%) of patients using injectable SRLs experience ongoing symptoms despite treatment (3, 162). Clinical and biochemical control may worsen at the end of the injection cycle: IGF-I concentrations were higher, PASQ scores worsened, and SRL concentrations were lower during the late vs early phase of the injection cycle (163, 164).

#### Oral therapies

Oral therapies may improve HRQoL by enhancing convenience, independence, and adherence. A phase 3 trial showed that acromegaly symptoms improved when patients switched from injectable octreotide to oral octreotide capsules (OOCs) (165), but a standardized QoL instrument was not used. MPOWERED, a randomized phase 3 trial, showed that OOC reduced treatment burden and improved convenience and satisfaction (166). Breakthrough symptoms were experienced in 31% vs 15% of the injectable vs oral octreotide group. Almost half (47%) of patients on injectable SRL reported injection site reactions, and 81% reported that the reactions interfered with daily activities (166). The open-label OOC extension showed improved treatment convenience, satisfaction, and symptom control (151).

Paltusotine, an oral somatostatin 2 receptor agonist, was approved by the US Food and Drug Administration (FDA) in 2025 for the treatment of adults with acromegaly who had an inadequate response to surgery and/or for whom surgery is not an option (167). ACROBAT Edge assessed safety and efficacy in patients switched from injectable SRLs to paltusotine (168), and 2 phase 3 randomized, placebo-controlled trials (PATHFNDR-1 and −2) (124, 169) used the ASD to assess patient symptoms (12). PATHFNDR-1 and PATHFNDR-2 showed statistically significant differences in total ASD scores, a prespecified secondary endpoint subject to formal hierarchical statistical testing, favoring the paltusotine group (124, 169).

HRQoL and mood outcomes are central to patients with acromegaly (170) and reflect biochemical control, symptom control, side effect profile, route of administration and its impact on the patient's time and autonomy, the complexity and severity of the underlying disease process, accessibility of the medication, and financial burden.

### Interpersonal and social well-being

Acromegaly exerts an ongoing impact on interpersonal relationships and social well-being, with both the disease and its treatments contributing to the burden. Cross-sectional and prospective studies using validated instruments show that interpersonal relations and social domains remain impaired even after successful treatment (22, 116, 125). In a patient meeting hosted by Acromegaly Community, nearly half of the patients reported difficulties in social interaction, and one-third reported strain in family relationships (110).

#### Disease-related factors

The chronic nature of progressive physical changes, comorbidities, and mood disorders contributes to impaired social functioning and strained relationships (117, 128, 130). Greater comorbidity burden and ongoing symptoms independently predict poorer social outcomes (116). Body image concerns, especially in women, can drive social withdrawal, sexual dysfunction, and reduced self-esteem (22, 130). Cognitive dysfunction and fatigue limit social participation and daily activities (54, 117).

#### Treatment-related factors

Acromegaly therapies improve morbidity and mortality but do not always enable full recovery of social well-being. Even after long-term biochemical control, patients report persistent symptoms and impaired HRQoL, particularly in the social and appearance domains (9, 22, 116, 125). Treatment burden with ongoing injections, including pain and loss of independence, can interfere with work, leisure, and social activities (116, 133). Life-long medical therapy associated with physical and psychological adverse effects, as well as time and financial burden, can strain caregivers and partners and limit social engagement.

#### Psychosocial determinants

Illness acceptance predicts HRQoL, including in social domains (171). Depression, anxiety, and body image concerns determine impaired social well-being (16, 128, 130). Early psychological intervention and multidisciplinary care are recommended to address persistent symptoms and psychosocial needs (16, 172).

## Activities of daily living, work productivity, and financial burden in acromegaly

Acromegaly imposes a significant burden on functional capacity, work productivity, and financial stability, even after patients achieve biochemical control (2, 172). Recognizing this multidimensional impact is essential for comprehensive management and health-economic planning. Many patients continue to experience functional limitations in walking, lifting, climbing stairs, or performing motor skill tasks due to joint pain, soft-tissue changes, and fatigue (16, 78, 115), impairing work productivity.

The work productivity and activity impairment questionnaire demonstrates both absenteeism and presenteeism in acromegaly (118, 173). One study found that 16% of patients reported repeated lost working days due to treatment burden (93). Among biochemically controlled patients, 92% reported symptom interference with daily life and 84% reported interference with leisure or work activities (118).

Impairments also adversely impact domestic and social situations. Patients report reduced participation in cleaning and shopping, and lower engagement in social or recreational events due to fatigue, joint pain, and physical limitations, despite disease control (174, 175). This disconnect between biochemical control and ongoing disease burden underscores the need for multidimensional outcome assessments that capture symptom and treatment burden on social participation (85).

The cumulative economic impact is considerable. Direct medical costs are several times higher than population averages (176, 177), reflecting multimodal care and treatment of related diabetes, hypertension, obstructive sleep apnea, and joint and cardiovascular disease (1, 2). Indirect costs arise from lost productivity, reduced income potential, reliance on caregivers, and early retirement (178).

### Determinants of financial burden and lost work productivity

Diagnostic delay, often 5 to 10 years (1, 179), contributes to economic and functional burden (180). During this period, progressive musculoskeletal changes and arthropathy (89) reduce mobility and employment opportunities. Patients experience psychosocial distress and diminished productivity before diagnosis. Diagnostic delay is also associated with larger adenomas presenting at diagnosis, higher comorbidity burden, and lower surgical remission rates (181), increasing the need for life-long pharmacotherapy. The FDA's externally led patient-focused drug development (PFDD) initiative highlights career disruptions, missed promotions, and caregiver reliance resulting from delayed diagnosis (182).

Even with effective treatment, ongoing symptom burden impairs productivity. Musculoskeletal pain and arthropathy restrict mobility and endurance. Fatigue, headaches, and sleep apnea contribute to impaired concentration and cognitive slowing (16, 183). Soft-tissue and acral changes impair fine motor skills such as typing, and frequent clinic visits for monitoring and testing add to time and cost burdens. These limitations result in absenteeism and presenteeism, and reduced household productivity (172, 184). Residual symptoms result in additional therapeutic interventions, further increasing financial burden and time investment (118).

### Impact of treatment on financial burden

#### Surgery

Transsphenoidal surgery is typically the first-line treatment, and if remission is achieved, the financial burden of lifelong pharmacotherapy is avoided. Initial hospital expenses in the US range from $20 000 to $45 000, depending on approach and complications (177, 185). Additional costs arise from time away from work and long-term hormone therapy required for hypopituitarism. Up to 60% of patients require lifelong multimodal therapy (1, 186), driving up cost.

#### Medical therapies

##### Injectable SRLs

###### Octreotide LAR

Monthly IM injection: US list price approximately $5500 to $6700 per injection ($66 000–$80 000 annually) (187). Monthly clinic visits add indirect costs through lost productivity and appointment fees.

###### Lanreotide depot

Monthly deep subcutaneous (SC) injection with the potential for self-injection. Annual costs are similar to those of octreotide, though self-injection may mitigate productivity loss (188).

###### Pasireotide LAR

Monthly IM injection: hyperglycemia in 35% to 45% of patients requires additional monitoring and therapy. Annual costs range from $204 000 to $240 000 (189, 190).

##### Growth hormone receptor antagonist

###### Pegvisomant

Daily SC injection: Depending on dose, annual expenditures exceed $150 000 (191). Regular liver function testing and injection burden add to the total cost (192).

##### Oral therapies

###### Cabergoline

Oral dopamine agonist: Annual costs range from $215 to $1460 annually, depending on dose (193).

###### Oral octreotide

Twice-daily dosing: annual costs are $78 000 to $96 000 (194). While eliminating the need for injections, food restrictions may affect adherence.

###### Paltusotine

Once-daily oral medication: costs are estimated at $290 000 annually.

Financial assistance is often available for patients to help manage the cost of brand-name medications.

##### Radiation therapy

Stereotactic or fractionated radiotherapy is considered for aggressive or refractory adenomas. Treatment costs in the US range from $25 000 to $40 000. Because biochemical remission may take years, patients incur pharmacotherapy costs during this time. Low remission rates imply that many patients remain on life-long medication (121, 122, 195-197). Approximately 80% of patients develop hypopituitarism at 10 years, adding costs for life-long medications, monitoring, multidisciplinary care, and complication management, as well as lost work productivity (122, 198).

##### Surveillance burden

Lifelong surveillance includes biochemical testing, serial magnetic resonance imaging scans, and monitoring of diabetes, hypertension, obstructive sleep apnea, musculoskeletal disease, and cardiovascular disease. Screening colonoscopies, thyroid ultrasound, cardiac echocardiography, and sleep studies add further costs (199, 200). Even when well-controlled, ongoing testing, lost work time, and caretaker costs contribute to economic and productivity burden.

### Strategies to improve the patient experience

Over the past 20 years, disease management has shifted from a primarily biochemical focus toward a more patient-centered approach. Despite surgical, pharmacologic, and disease monitoring advances, many patients continue to experience persistent symptoms, psychosocial burden, and impaired QoL. Improving the patient experience requires integrated strategies that address both medical and nonmedical dimensions of care, combining therapeutic innovation, multidisciplinary collaboration, mental health support, and individualized treatment goals and ensuring that patient perspectives inform clinical practice and regulatory policy (170).

#### Advances in therapeutic options

Expanded treatment options reduce some of the burden associated with injectable regimens. Long-acting SRLs, lanreotide depot, and octreotide LAR, remain foundational, but newer formulations and longer duration delivery systems aim to minimize injection frequency, improve adherence, and reduce treatment fatigue. Ready-to-use, SC depot octreotide formulation for monthly self-administration via a prefilled autoinjector pen has shown promising efficacy with improved IGF-I control, symptom scores, and PROs (201).

Oral therapies OOC and paltusotine (166, 168) represent a paradigm shift in management, providing noninvasive alternatives for appropriately selected patients and highlighting the importance of therapeutic convenience in long-term disease control. Longer duration injectables and sustained-release implants may further promote patient autonomy. Collectively, these innovations reflect a therapeutic landscape increasingly shaped by patient preference and lifestyle considerations.

#### Patient-centered and multidisciplinary care

Coordinated, multidisciplinary collaboration among endocrinologists, neurosurgeons, neuroradiologists, radiation oncologists, cardiologists, sleep specialists, gastroenterologists, neuro-ophthalmologists, and mental health professionals is optimal. Comprehensive care includes monitoring of cardiovascular disease, diabetes, sleep apnea, and musculoskeletal complications. Multidisciplinary team meetings support integrated care planning, timely referrals, and coordinated follow-up.

Integration of patient-centered, validated PROs aligns therapeutic strategies with outcomes that matter most to patients. When such goals are incorporated into regular assessments, patients are more likely to remain engaged, adherent, and empowered in their management. Incorporating PRO data into electronic health records and registries may also help identify unmet needs.

#### Shared decision-making and individualized treatment goals

Shared decision-making is central to acromegaly care, where treatment options differ by administration route, side effect profile, monitoring requirements and cost. Clinicians should discuss treatment expectations, injection burden, travel constraints, and fertility considerations to ensure that patients’ preferences guide the treatment selection. Treatment goals should be co-defined and revisited regularly to capture evolving priorities as the disease and management of the disease progress.

#### Role of patient advocacy groups

Patient advocacy groups play a meaningful role in acromegaly care. Beyond psychosocial support, they help connect patients, clinicians, and policymakers and contribute to the incorporation of the patient voice in drug approvals and regulatory discussions. Advocacy groups also lead to improved awareness of specialized resources, including high-volume pituitary centers and allied specialists familiar with acromegaly, such as physical therapists, physiatrists, pain specialists, orthopedists, and mental health counselors. Advocacy groups increase awareness of clinical trials and encourage participation, with the potential to support research progress and offer patients optimal care opportunities. Educational initiatives led by advocacy groups promote shared decision-making and patient empowerment, leading to improved outcomes and satisfaction. Further study is needed to better define the effects of advocacy group involvement on patient care, disease burden, research participation, and in enabling improved clinical outcomes.

#### Policy, advocacy, and regulatory pathways to amplify the patient voice

Systemic strategies integrate patient perspectives in drug development and regulatory decision-making. The FDA's PFDD program and similar initiatives globally capture patient experiences, priorities, and risk tolerance. The PFDD meeting for acromegaly, organized with the Acromegaly Community and other stakeholders, exemplifies how structured engagement can influence regulatory science, clinical trial design, endpoint selection, and labeling, ensuring that future therapies address disease dimensions most impactful to patients.

## Future directions

Persistent QoL impairment underscores the need for comprehensive, patient-centered definitions of treatment success that incorporate symptom control, daily functioning, and overall well-being. To optimize patient-centered care, clinicians should routinely use validated PRO instruments, interpret changes relative to MID thresholds, and address comorbidities and persistent symptoms alongside biochemical parameters.

Future research should include standardized, validated PRO measures in clinical trials. Head-to-head comparative studies should be conducted, and MID thresholds for PROs should be validated to improve clinical relevance. Availability of rigorous consensus core outcome sets, including biochemical control, adenoma mass control, symptom burden, HRQoL, mood, physical functioning, treatment satisfaction, and work productivity, will all contribute to enhanced evidence-based management. Standardized longitudinal assessment of these domains in clinical trials and observational studies would improve comparability across studies and provide a more patient-centered definition of treatment success. Use of validated PRO measures within this framework would help ensure consistent capture of outcomes most relevant to patients.

Acromegaly management should define success as biochemical control plus QoL restoration, requiring multidisciplinary collaboration, consistent patient participation, and shared decision-making.

## Conclusions

Acromegaly is associated with substantial symptom burden, impaired QoL, and unmet psychosocial needs, even among biochemically controlled patients. Comprehensive patient-centered care should address persistent symptoms, psychological health, and functional limitations. Multidisciplinary expertise and patient engagement are essential for achieving optimal long-term outcomes.

## Data Availability

Data sharing is not applicable to this article as no data sets were generated or analyzed during the present study.

## Abbreviations

AcroQoL: Acromegaly Quality of Life Questionnaire
Acro-TSQ: Acromegaly Treatment Satisfaction Questionnaire
ASD: Acromegaly Symptom Diary
BDI: Beck Depression Inventory
EQ-5D: EuroQol 5 dimension
FDA: US Food and Drug Administration
GH: growth hormone
HRQoL: health-related quality of life
IGF-I: insulin-like growth factor 1
IM: intramuscular
LAR: long-acting release
MID: minimal important difference
OOC: oral octreotide capsules
PASQ: Patient-Assessed Acromegaly Symptom Questionnaire
PFDD: patient-focused drug development
PGWBS: psychological general well-being schedule
PRO: patient-reported outcome
PROMIS: patient-reported outcomes measurement information system
QoL: quality of life
SC: subcutaneous
SF: short form
SMD: standardized mean difference
SRL: somatostatin receptor ligand
